# Association between left ventricular global longitudinal strain and natriuretic peptides in outpatients with chronic systolic heart failure

**DOI:** 10.1186/s12872-015-0063-8

**Published:** 2015-08-20

**Authors:** F Gaborit, H Bosselmann, N Tønder, K Iversen, T Kümler, C Kistorp, G Sölétormos, J P. Goetze, M Schou

**Affiliations:** Department of Cardiology, Herlev University Hospital, DK-2730 Herlev, Denmark; Department of Internal Medicine KNEA, North Zealand University Hospital, DK-3400 Hillerod, Denmark; Department of Internal Medicine O, Herlev University Hospital, DK-2730 Herlev, Denmark; Department of Clinical Biochemistry, North Zealand University Hospital, DK-3400 Hillerod, Denmark; Department of Clinical Biochemistry, Rigshospitalet, DK-2100 Copenhagen, Denmark

**Keywords:** Left ventricular global longitudinal strain, Systolic heart failure, Natriuretic peptides

## Abstract

**Background:**

Both impaired left ventricular (LV) global longitudinal strain (GLS) and increased plasma concentrations of natriuretic peptides(NP) are associated with a poor outcome in heart failure (HF). Increased levels of NP reflect increased wall stress of the LV. However, little is known about the relationship between LV GLS and NP. This aim of this study was to evaluate the relationship between the echocardiographic measure LV GLS and plasma levels of NP.

**Methods:**

We prospectively included 149 patients with verified systolic HF at the baseline visit in an outpatient HF clinic. LV GLS was assessed by two dimension speckle tracking and plasma concentrations of N-terminal-pro-brain-natriuretic-peptide (NT-proBNP) and pro-atrial-natriuretic-peptide (proANP) were analysed.

**Results:**

The patients had a median age of 70 years, 28.2 % were females, 26.5 % were in functional class III-IV, median left ventricular ejection fraction (LVEF) was 33 % and median LV GLS was −11 %. LV GLS was associated with increased plasma concentrations of NT-proBNP and proANP in multivariate logistic regression (NT-proBNP: Odds Ratio_GLS_: 7.25, 95 %-CI: 2.48-21.1, P < 0.001 and proANP: Odds Ratio_GLS_: 3.26, 95-%-CI: 1.28-8.30, *P* = 0.013) and linear regression (NT-proBNP: β_GLS_: 1.19, 95 %-CI: 0.62-1.76, P < 0.001 and proANP: β_GLS_: 0.42, 95-%-CI: 0.11-0.72, *P* = 0.007) models after adjustment for traditional confounders (age, gender, body-mass-index, atrial fibrillation, renal function) and left atrial volume index.

**Conclusion:**

Impaired LV GLS is associated with increased plasma concentrations of NP and our data suggest that left ventricular myocardial mechanics estimated by LV GLS reflects myocardial wall stress in chronic systolic HF.

## Background

Left ventricular (LV) global longitudinal strain (GLS) is a novel echocardiographic method to evaluate LV function. It is more sensitive to LV dysfunction than conventional methods, and strain analysis allows for both regional and global assessment of the LV [1, 2]. GLS is reported as a negative value in percentages. A value closer to cero is a sign of impaired function of the LV. The normal range has been explored in some studies and it is reported to be in the region from −15.9 % to −22.1 %. The variation may be due to age, blood pressure and technical circumstances [3, 4]. It has been observed that LV GLS is associated with an increased risk of mortality and cardiovascular events in patients with established cardiac disease [5].

Among patients presenting with acute myocardial infarction, LV GLS is associated with development of heart failure (HF) and an increased mortality risk [6] as well as increased plasma concentrations of aminoterminal pro-brain natriuretic peptide(NT-proBNP) [7]. In chronic HF it has been shown that LV GLS has additional prognostic value and it can predict hospitalization for HF and an increased mortality risk [8–10]. However it remains unknown whether LV GLS reflects e.g., myocardial wall stress [11], fibrosis [12] or subclinical ischemia [13] in chronic HF patients. NT-proBNP is released from the myocardium when it is exposed to stretch and increased wall stress [14]. Only few data exist on the relationship between LV GLS and NT-proBNP in patients with HF, suggesting association between impaired LV GLS and increased NT-proBNP [15]. Among patients suspected of HF LV GLS has been reported to be a strong predictor of NT-proBNP [16]. In asymptomatic patients with echocardiographic verified diastolic dysfunction GLS rate showed the strongest correlation to increased NT-proBNP [17].

In the present study we, therefore, tested the hypothesis that impaired LV GLS is associated with increased plasma concentrations of NT-proBNP and pro-atrial natriuretic peptide (proANP) in chronic systolic HF patients.

## Methods

### Patient selection

Patients were enrolled prospectively at their first visit when referred to the outpatient HF clinic at North Zealand Hospital, University of Copenhagen, Denmark. From January 2011 to November 2012, *N* = 230 patients were referred to the HF clinic with LVEF < 45 %. Patients had to be clinically stable (60 days out of hospital) and have steady state plasma-creatinine concentration for 60 days (+/−10 μg). A total of *N* = 149 patients agreed to participate in an echocardiography study (Cardio-Ren) [18]. All patients provide written informed consent according to the Declaration of Helsinki and the study was approved by the Scientific Ethic Committee (H-1-2010-074).

At baseline all patients were examined by a physician and the following information were obtained: medical history, present symptoms, functional class, medication, height, weight, non-invasive blood pressure, heart rate, 12-lead electrocardiogram. Venous blood samples were taken and advanced echocardiography was preformed according to standard recommendations of the European Society of Cardiology [19, 20].

### Echocardiography

Echocardiography was performed on Vivid 9E (GE Vingmed Ultrasound, Horten, Norway) and analysed offline (Echopac BT 12, 1.0, General Electric, Horten, Norway). All analysis was performed by a single operator blinded to plasma concentrations of NT-proBNP and proANP.

LVEF were determined using the Simpson biplane model. Two dimensional parasternal view were used to measure LV dimension and wall thickness. LV mass were calculated automatically by Echopac software from the linear dimensions and reported indexed to body surface area. Maximal left atrial volume were determined from the biplane area method and indexed to body surface area. Mitral inflow was evaluated by doppler recordings and peak velocity of early (E) and atrial filling (A) and mitral valve deceleration time were measured. Pulsed wave velocity was measured at both lateral and septal mitral annulus for myocardial peak early velocity (e’) and peak systolic velocity (s’). Mean e’ was calculated from the lateral and septal e’, and used for calculating E/e’. In case of atrial fibrillation the mean value of e’ from three consecutive cycles was used.

GLS analysis were performed by two-dimensional speckle tracking in the three standard apical projections (long axis, four chambers, two chambers). In the echocardiographic examination we aimed for a frame rate 40–80 frames/sec in all images for strain analysis. The region of interest was set to cover the thickness of the myocardium. Appropriate tracking were verified visually and adjusted if necessary. Aortic valve closure were automated in the analyse and then confirmed visually, when uncertain it was identified with continuous wave doppler in the aortic annulus. LV GLS were analysed for 17 standardised segments, based on these values a mean value were calculated for each of the three apical projections and then a total LV GLS were calculated as the average of the value from the three apical projections. The Echopac software allows up to one missing segment in each projection. If two segment values were missing in one projection we calculated the missing score as the average of neighbouring segments and then calculated LV GLS manually. LV GLS analysis could not be performed when three or more segments were missing over all or two segments were missing in more projections. Reproducibility were tested in 25 random selected patients for both inter- or intra observer variation. No significant bias were found (intra observational: mean diff: −0.08 % +/− 1.16 %, *P* = 0.703; inter observational: mean diff: 0.19 % +/−1.9 %, *P* = 0.422).

### Plasma analyses

Venous blood were obtained after >8 hours overnight fast and 15 min rest. At the time of sampling P-hemoglobin, P-creatinine, P-sodium and P-potassium were measured. For later analyses plasma were collected in ethylenediamine tetracetic (EDTA) vial and centrifuged at 4° (3000 rpm in 10 min) and stored at −80° Celsius. Plasma concentrations of NT-proBNP were measured on the Dimension Vista® 1500 from Siemens Medical Solutions Diagnostics using the LOCI®-technology (Luminescent Oxygen Channeling Assay) according to the manufacturer procedure [21]. Total proANP was measured with a processing-independent radioimmunoassay, which quantitates the total sum of unprocessed and processed N-terminal proANP fragments [22].

### Statistics

Baseline clinical data, biochemical data (Table 1) and echocardiographic measures (Table 2) are presented as percentages for dichotomous variables and interquartile range (25 %-75 %) for continuous variables. Groups were compared with Pearson Chi square-test for discrete variables and t-tests (parametric) and Mann Whitney U-tests (nonparametric) for continuous variables, as appropriate. Multivariate and univariate regression analyses were made test the relationship between LV GLS and NT-proBNP or proANP. Both linear and logistic regression analyses were tested (Tables 3 and 4). Adjustments were made for established confounders: gender, age, body mass index (BMI), atrial fibrillation, left atrial size and plasma eGFR. Additional univariate regression analyses were used to crosscheck the relationship between the ability to obtain sufficient image quality for LV GLS analysis and the co-existing of obesity (BMI >35 kg/m2) or chronic obstructive pulmonary disease (no significant relationship were observed). Statistic results was considered significant when P < 0.05 (two-sided).

**Table 1:** Baseline characteristics according to values below median (more negative) (*n* = 65) or above median (more positive) (*n* = 52), median GLS (median = −11 %)

Variable	Below median GLS	Above median GLS	p-value
	(*n* = 65)	(*n* = 52)	
Age, years	69 (63–76)	71 (67–76)	0.761
Female gender, %	33.8	21.2	0.130
BMI^a^, kg/m2	26.0 (22.6-30.2)	26.3 (24.5-30.3)	0.214
NYHA^b^ class III-IV, %	23.1	30.8	0.349
Systolic BP^c^, mmHg	132 (120–147)	120 (110–140)	0.178
Diastolic BP^c^, mmHg	77 (70–82)	77 (69–84)	0.889
Heart rate, beats/min	64 (56–76)	70 (62–78)	0.188
HF^d^ duration, months	9 (6–12)	12 (6–24)	0.216
Pervious MI^e^, %	33.8	44.2	0.251
Hypertension, %	67.7	59.6	0.395
Atrial fibrillation, %	24.6	40.4	0.068
ICD^f^, %	9.5	13.5	0.507
Diabetes, %	20	25	0.518
ApoplexiaCerebri/TCI^g^, %	12.3	23.1	0.124
ACE-I^h^, %	73.8	59.6	0.140
ARB^i^, %	21.5	32.7	0.174
Beta-blocker, %	87.7	86.5	0.853
MRA^j^, %	14.1	28.8	0.051
Diuretics, %	69.2	76.9	0.354
Hemoglobine, mmol/l	8.5 (7.0-9.0)	8.8 (8.0-9.3)	0.215
Creatinine, umol/l	80 (68–97)	95 (73–119)	0.254
eGFR^k^, ml/min/1,73 m2	82 (66–98)	70 (51–95)	0.244

^a^Body mass index, ^b^New York Heart Association, ^c^Blood pressure, ^d^Heart failure, ^e^Myocardial infarction, ^f^Implantable cardiac defibrillator, ^g^Transitory cerebral ischemia, ^h^Angiotensin converting enzyme inhibitor, ^i^Aldosterone receptor blocker, ^j^Mineralocorticoid receptor antagonist, ^k^Estimated glomerular filtration rate

**Table 2:** Echocardiographic variable according to values below or above median GLS, with 75 % interquartile range

Variable	Below median GLS	Above median GLS	p-value
	(*n* = 65)	(*n* = 52)	
LVEF biplane^a^, %	38 (32–42)	27 (20–34)	<0.001
LVIDd^b^, cm	5.00 (4.55-5.55)	5.70 (5.20-6.30)	<0.001
Peak GLS^c^	−13 (−15 - -12)	−8 (−10 - -6)	<0.001
TAPSE^d^, mm	19.0 (15.0-23.0)	16.0 (14.0-20.8)	0.012
TRmax^e^,mmHG	22.52 (8.48-27.25)	16.98 (4.28-26.70)	0.474
s’ medial^f^, cm/s	5.00 (4.00-6.00)	4.00 (3.00-5.00)	<0.001
s’ lateral^f^, cm/s	6.00 (5.00-7.00)	5.00 (4.00-5.00)	0.001
LAVI^g^, ml/m2	27.09 (21.18-33.00)	31.57 (23.17-39.10)	0.093
MV deceleration time^h^, ms	222 (178–277)	182 (152–223)	0.027
E^i^/A^j^ ratio	0.89 (0.67-1.36)	0.86 (0.62-1.65)	0.937
E^f^/e’^k^	9.68 (7.53-12.20)	10.47 (7.75-15.27)	0.130
LV mass Index, g/m2	123.60 (90.85-153.18)	145 (117.85-194.90)	0.019

^a^Left ventricle ejection fraction, ^b^Left ventricle internal dimension diastole, ^c^Peak left ventricle global longitudinal strain, ^d^Tricuspid annular plane systolic excursion, ^e^Maximal pressure gradient of tricuspid regurgitation, ^f^Myocardial peak systolic velocity, ^g^Left atrial end systolic volume index, ^h^Mitral valve, ^i^Mitral inflow early filling, ^j^Mitral inflow atrial filling, ^k^Myocardial early velocity

**Table 3:** Univariate and multivariate logistic regression models

Variable	Univariate		Multivariate	
	Odds ratio	Beta	Odds ratio	Beta
	(95 % CI^a^)	(95 % CI)	(95 % CI)	(95 % CI)
	P-value	P-value	P-value	P-value
GLS^b^_ NT-proBNP_^c^	5.92	0.47	7.25	1.19
	(2.59-13.60)	(0.15-0.32)	(2.48-21.18)	(0.62-1.76)
	<0.001	<0.001	<0.001	<0.001
GLS_Age_	-	-	1.03	0.03
			(0.97-1.09)	(0.00-0.06)
			0.310	0.025
GLS_GenderFemale_	-	-	2.29	0.07
			(0.62-8.47)	(−0.55-0.69)
			0.214	0.822
GLS_ BMI_^d^	-	-	0.89	−0.05
			(0.80-1.00)	(−0.10-0.00)
			0.047	0.063
GLS_ AF_^e^	-	-	3.05	0.52
			(0.94-0.85)	(−0.10-1.14)
			0.063	0.099
GLS_Creatinine_	-	-	1.03	0.01
			(1.00-1.05)	(0.00-0.01)
			0.010	<0.001
GLS_ LAVI_^f^	-	-	1.00	0.00
			(0.99-1.02)	(−0.01-0.01)
			0.647	0.569

^a^Confidence interval, ^b^Global longitudinal strain, ^c^Amino-terminal-pro-brain-natriuretic-peptide, ^d^Body mass index, ^e^Atrial fibrillation, ^f^Left atrial volume index

Confounders included in the multivariate logistic regression models: Age, gender, atrial fibrillation, body-mass-index, creatinine, LAVI

A: Univariate and Multivariate logistic regression models (response: NT-proBNP) (confounders: age, gender, body mass index, atrial fibrillation, creatinine, left atrial volume index)

**Table 4:** Univariate and multivariate logistic regression models

Variable	Univariate		Multivariate	
	Odds ratio	Beta	Odds ratio	Beta
	(95 % CI^a^)	(95 % CI)	(95 % CI)	(95 % CI)
	P-value	P-value	P-value	P-value
GLS^b^_proANP_^c^	3.37	0.34	3.26	0.42
	(1.53-7.40)	(0.05-0.16)	(1.28-8.30)	(0.11-0.72)
	0.003	<0.001	0.013	0.007
GLS_Age_	-	-	1.05	0.05
			(1,00-1.11)	(0.03-0.06)
			0.075	<0.001
GLS_GenderFemale_	-	-	1.18	-.023
			(0.39-3.58)	(−0.55-0.10)
			0.773	0.172
GLS_ BMI_^d^	-	-	0.96	−0.02
			(0.88-1.05)	(−0.05-0.00)
			0.402	0.097
GLS_ AF_^e^	-	-	0.80	−0.05
			(0.28-2.29)	(−0.38-0.28)
			0.671	0.746
GLS_Creatinine_	-	-	1.02	0.00
			(1.00-1.04)	(0.00-0.01)
			0.030	<0.001
GLS_ LAVI_^f^	-	-	1.00	0.00
			(0.99-1.01)	(0.00-0.01)
			0.960	0.245

^a^Confidence interval, ^b^Global longitudinal strain, ^c^pro-atrial-natriuretic-peptide, ^d^Body mass index, ^e^Atrial fibrillation, ^f^Left atrial volume index

Confounders included in the multivariate logistic regression models: Age, gender, atrial fibrillation, body-mass-index, creatinine, Left atrial volume index

B: Univariate and Multivariate logistic regression models (response: proANP)(Confounders: age, gender, body mass index, atrial fibrillation, creatinine, left atrial volume index)B: Univariate and Multivariate logistic regression models (response: proANP)(Confounders: age, gender, body mass index, atrial fibrillation, creatinine, left atrial volume index)

## Results

We were able to perform LV GLS analysis in 117 of 149 patients, corresponding to 80 %, and median LV GLS was −11 %. Ischemic heart disease determined by coronary angiography was present in 58 % of the patients, and 38 % of all patients had atrial fibrillation. Baseline characteristic are presented in Table 1 according to LV GLS below (more negative) or above (more positive = impaired) median LV GLS. There were no significant differences between baseline characteristics in the two groups.

In Table 2 echocardiographic variables are presented. Patients with a LV GLS above the median had lower LVEF (P < 0.001), mildly enlarged LV internal dimension in diastole (P < 0.001) and lower s’ both laterally (P < 0.001) and medially (P < 0.001). Diastolic function estimated by mitral valve deceleration time (*P* = 0.027) was reduced in patients with LV GLS above the median, but left atria size and E/e’ were not significantly affected by LV GLS. Right ventricular systolic function estimated by TAPSE (*P* = 0.012) was also reduced in patients with LV GLS above the median. LV mass index was significantly higher in patients with a GLS above the median (*P* = 0.019).

**Fig. 1:**
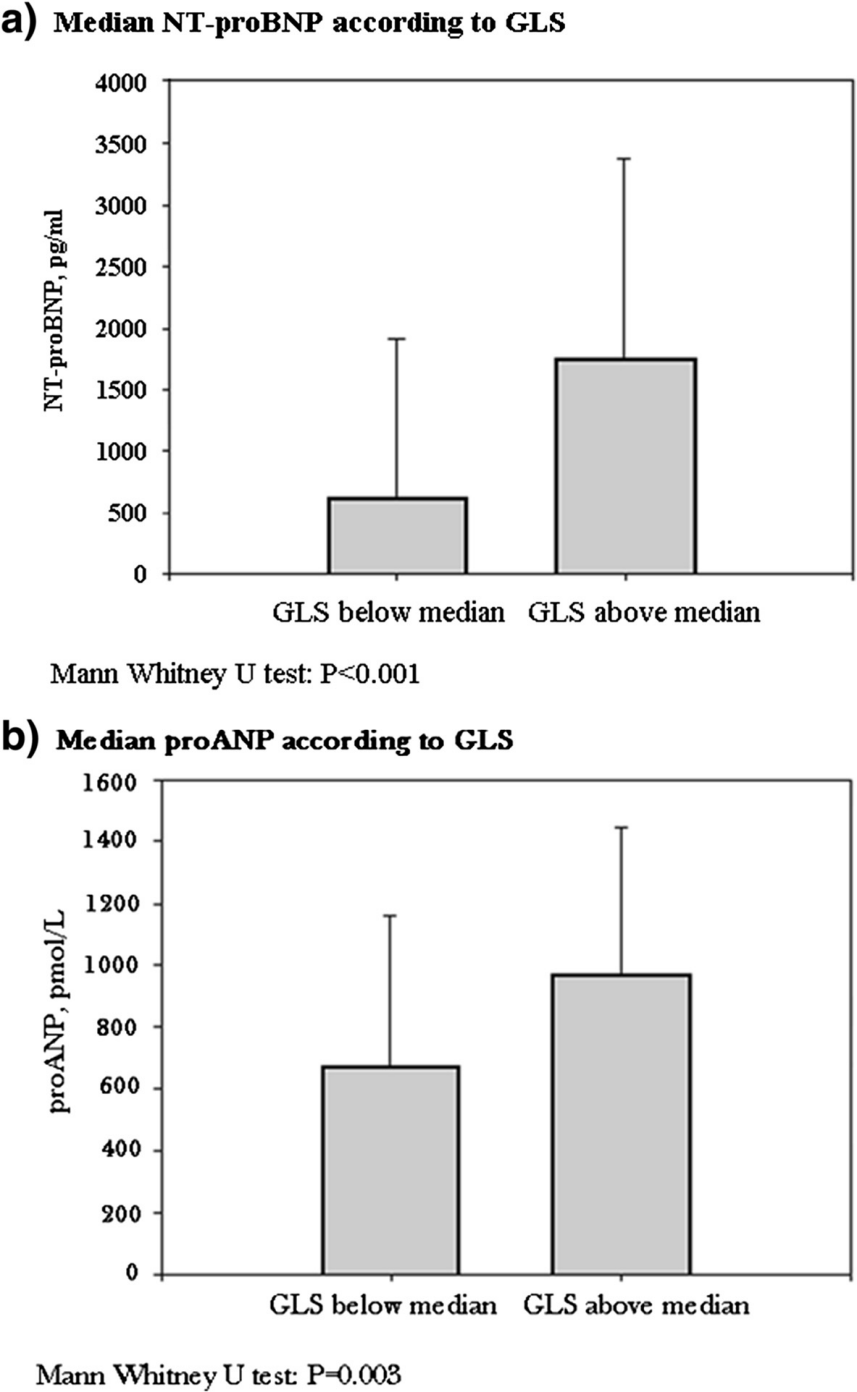
**a** Median NT-proBNP according to GLS above or below median, with 75 % interquartile range. **b** Median proANP according to global longitudinal strain above or below median, with 75 % interquartile range. GLS: global longitudinal strain; NT-proBNP: amino-terminal-pro-brain-natriuretic-peptide; proANP: pro-atrial-natriuretic-peptide

**Fig. 2:**
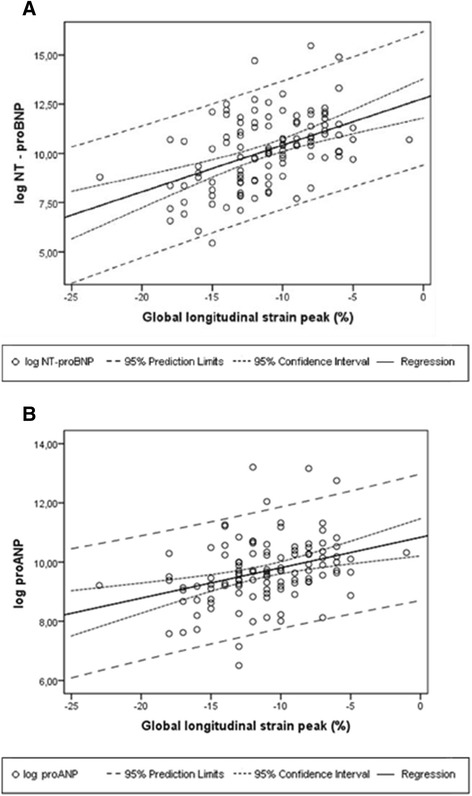
**a** Correlation between NT-proBNP and GLS. **b** Correlation between MR-proANP and global longitudinal strain. GLS: global longitudinal strain; NT-proBNP: amino-terminal-pro-brain-natriuretic-peptide; proANP: pro-atrial-natriuretic-peptide

Plasma concentrations of NT-proBNP and proANP were significantly higher in patients with LV GLS above the median (Fig. 1). The overall linear relationship between LV GLS and log(NT-proBNP) and LV GLS and log(proANP) are shown in Fig. 2. In multivariate linear regression models with log(NT-proBNP) and log(proANP) as response variables and LV GLS as explanatory variable the associations were significant between LV GLS and log(NT-proBNP) (β_(NT-proBNP)_ = 1.19 (95 %-CI: 0.62-1.76, p < 0.001) and LV GLS and log(proANP) (β_(proANP)_ = 0.42, (95 %-CI: 0.11-0.72, *P* =0.007). The associations between impaired LV GLS and log(NT-proBNP), and LV GLS and log(proANP), respectively, were also significant in multivariate logistic regression models (Odds Ratio GLS_NT-proBNP_ 7.25 (95 %-CI: 2.48-21.18, P < 0.001) and (Odds Ratio GLS_proANP_ 3.26 (95 %-CI: 1.28-8.29, *P* = 0.013).

## Discussion

The main finding of this study is that impaired LV GLS is associated with increased cardiac wall stress expressed by elevated plasma concentrations of NT-proBNP and proANP in chronic systolic HF patients.

### Echocardiographic findings and GLS

In the present study of patients with chronic systolic HF the majority of patients had impaired LV GLS. Patients with more impaired LV GLS had lower LVEF, and moreover, LV GLS showed stronger correlation to echocardiographic variables related to systolic function than to diastolic function. Previous studies have observed same association between LV GLS and systolic function [5, 8, 10]. LV GLS has also been evaluated in patients with HF and preserved LVEF and these studies have also identified LV GLS to be associated with systolic function and not conventional echocardiographic measures of diastolic dysfunction [23, 24]. These previous findings, including our result, therefor suggest that LV GLS reflects systolic function of the LV in both HF with reduced and normal LVEF.

### Cardiovascular Biomarkers and GLS

Few studies have examined LV GLS and NP in chronic systolic HF. In a study of 137 patients with suspected HF Yoneyama et al. [16] observed that plasma concentrations of brain-natriuretic-peptide (BNP) were correlated to functional class, decreased LVEF and an impaired GLS in patients with both systolic and diastolic dysfunction. This is supported by Nahum et al. who examined 125 patients with chronic systolic heart failure and found not only prognostic value of LV GLS, but also association between both GLS and NT-proBNP and strain rate and NT-proBNP [15]. In patients with acute myocardial infarction Ersbøll et al. observed that impaired LV GLS was correlated to plasma concentrations of NT-proBNP [7] and risk of HF during the admission [25]. The previous and the present studies, therefore, suggest that LV GLS reflects wall stress in HF patients since plasma concentration of BNP and NT-proBNP reflect myocardial wall stress [10]. It should be noted that both LV GLS and natriuretic peptides may be indirect measures of myocardial wall stress and future studies should evaluate the relationship between wall stress based on invasive measures and LV GLS and plasma concentrations of NP’s before any firm conclusions can be made. Whether impaired LV GLS also reflects other aspects than increased wall stress in chronic systolic HF needs to be investigated in future biomarker studies including e.g., Galectin 3.

### Methodological considerations and perspectives

Some methodological limitations of this study should be addressed. It is a single center study and consists of a relatively small cohort. Patients in this study have a short history of chronic systolic HF, are in stable clinical condition over past tree month and may not be fully up-titrated in the recommended HF treatment. Therefore the results cannot be extrapolated to other groups of patients or other stages of heart failure.

All patients had performed an echocardiography by a trained physician. Due to poor acoustic conditions or atrial fibrillation we were not able to perform LV GLS analyses in 21.5 % of the patients. Atrial fibrillation is a challenge when LV GLS is analysed, because of variation in the electrocardiographic loop duration, but if cycles of same length are selected, corresponding to a normal heart rate. LV GLS can be calculated in patients with atrial fibrillation [26]. It may be speculated that patients with atrial fibrillation more often have an impaired LV GLS since atrial fibrillation is associated with increased plasma concentrations of NP’s [27], but why NP’s are increased in atrial fibrillation is unknown, and exclusion of some patients with atrial fibrillation will probably not result in selection bias. In the present study the prevalence of atrial fibrillation did not differ significantly between the two groups of LV GLS above and below the median (Table 3). A BMI > 35 kg/m2 and chronic obstructive lung disease (prevalent conditions in HF) did neither influence the echocardiographic image quality and it was possible to analyse LV GLS in these subgroups. Selection bias due poor echocardiographic quality in certain subgroups seems, therefore, not to be the case.

Calculation of LV GLS is not yet a standard procedure in the echocardiographic examination. The challenge of LV GLS is that it requires good imaging quality of all three apical standard projections and sinus rhythm is preferable. LV GLS is only slightly angle dependent [28]. The calculation of LV GLS is automatic after setting the region of interest and intra- and interobserver variation is low. Theoretically, if LV GLS is going to be analysed in clinical practice it may be possible to find patients at high risk of developing decompensating and worsening of HF, and an hospitalization might be prevented if a relevant intervention is applied, but this hypothesis needs to be validated in a prospective setting.

## Conclusion

Impaired LV GLS is associated with increased plasma concentrations of NP’s and patients with impaired LV GLS have more frequent other cardiac abnormalities and diastolic and systolic dysfunction. GLS of the LV is, therefore, both associated with other structural abnormalities of the heart and neurohormonal activity. LV GLS is an accessible echocardiographic meassure and should be used in future studies for risk stratification and to identify clinical or scientific scenarios where GLS might be more useful than NP’s.

## Abbreviations

LV: Left ventricle
GLS: Global longitudinal strain
NP: Natriuretic peptide
HF: Heart failure
NT-proBNP: Aminoterminal pro-brain natriuretic peptide
ProANP: Pro-atrial natriuretic peptide
LVEF: Left ventricle ejection fraction
EDTA: Ethylenediamine tetracetic
NYHA: New York Heart Association
BMI: Body mass index
eGFR: Estimated glomerular filtration rate
